# One-Year Evaluation of High-Power Rapid Curing on Dentin Bond Strength

**DOI:** 10.3390/ma17102297

**Published:** 2024-05-13

**Authors:** Eva Klarić, Josipa Vukelja Bosnić, Matej Par, Zrinka Tarle, Danijela Marovic

**Affiliations:** 1 Department of Endodontics and Restorative Dentistry, University of Zagreb School of Dental Medicine, 10000 Zagreb, Croatia; eklaric@sfzg.hr (E.K.); mpar@sfzg.hr (M.P.); tarle@sfzg.hr (Z.T.); 2 Private Dental Practice, 10000 Zagreb, Croatia; jvukelja@sfzg.hr

**Keywords:** shear bond strength, rapid curing, high-intensity curing, bulk-fill, composites, universal adhesive

## Abstract

This study investigated the effect of 3 s light-curing with a high-power LED curing unit on the shear bond strength of bulk-fill composites. Four bulk-fill composites were bonded to dentin with a universal adhesive (Scotchbond Universal Plus): two materials designed for rapid curing (Tetric PowerFill and Tetric PowerFlow) and two controls (Filtek One Bulk Fill Restorative and SDR Plus Bulk Fill Flowable). The 4 mm composite layer was light-cured with Bluephase PowerCure for 20 s at 1000 mW/cm^2^ (“20 s”) or for 3 s at 3000 mW/cm^2^ (“3 s”). The samples were stored at 37 °C in distilled water and tested after 1, 6 and 12 months. The samples polymerised in the “3 s” mode had statistically similar or higher bond strength than the samples cured in “20 s” mode, except for the Tetric PowerFlow (1 month) and SDR+ (6 month). The flowable materials Tetric PowerFlow and SDR Plus initially showed the highest values in the “3 s” and “20 s” groups, which decreased after 12 months. The bond strength was statistically similar for all materials and curing protocols after 12 months, except for Tetric PowerFill cured with the “3 s” protocol (21.22 ± 5.0 MPa), which showed the highest value. Tetric PowerFill showed the highest long-term bond strength. While “3 s” curing resulted in equal or better shear bond strength, its use can only be recommended for a material with an AFCT agent such as Tetric PowerFill.

## 1. Introduction

The combination of the mechanical interlocking, chemical and physical forces that indicate the molecular attraction between materials in close contact is known as adhesion. In dentistry, this can be accomplished by using the correct primers, bonding, cements, and composite materials in addition to properly preparing the joined surfaces chemically (by acid etching) and physically (by grinding and sandblasting) [1]. Adhesion is the cornerstone of modern restorative dentistry.

However, the dentist’s desire to simplify and shorten the clinical procedure of placing a composite restoration could compromise adhesion. This desire has prompted manufacturers to respond with a fast curing time of 3 s by using a powerful 3000 mW/cm^2^ light source. Universal adhesives, the latest developments in dental adhesive technology, are also part of this trend to maximise the efficiency of the practitioner and facilitate application in different situations. Universal adhesives can be used in full etch-and-rinse, selective enamel etch, or self-etch bonding mode [2]. Moreover, they offer a range of applications and the capacity to adhere to indirect tooth restorations such as zirconia ceramics with and without glass [3,4].

One of the most common criticisms of universal adhesives is that its thin film, which is frequently less than 10 µm, allows oxygen to inhibit polymerization over a significant portion of its depth. Suboptimal polymerization leads to an insufficient stabilization of the adhesive interface, reducing the ability of the adhesive layer to resist stresses. There is another factor that can affect bond strength and is not often considered in research: the storage of the bonding agent and its service life are considered key components in the phenomenon of extraoral degradation of adhesive systems. It should be kept in mind that problems related to the preservation of the adhesion system can compromise the efficiency and stability of adhesion to the dental substrate [5].

In addition to adhesives, currently available composites have undergone some modifications. Most photoinitiators are triggered by visible light radiation that falls within the blue spectral range. The most common photoinitiator found in most commercial products, camphorquinone, absorbs wavelengths between 410 and 500 nm, with a maximum absorption at 468 nm. However, due to its strong yellow hue, manufacturers have switched to brighter alternatives (such as phenylpropanedione, Lucirin TPO, and Ivocerin, a diacylgermane-based photoinitiator), which are activated by lower wavelengths in the violet spectrum (370–460 nm) [6]. 

In addition to a more favourable colour, one of the more effective Norrish type I sensitizers is Ivocerin (Ivoclar, Schaan, Liechtenstein), an alternative photoinitiator based on diacylgermane that cleaves into two photoradicals after exposure to radiation of a specific length. When a tertiary amine is added as a co-initiator to the traditional photoinitiator, camphorquinone, a Norrish type II sensitizer, it can be photoactivated and generate a single radical [7]. Furthermore, Ivocerin is more active than the camphorquinone–amine system [8] at wavelengths of about 410 nm due to its greater molar extinction coefficient [9]. Photoactivation is reportedly more effective when cured with the blue–violet spectrum [6], despite reports that some dual-cure resin cements can only be cured with blue light [9]. Ivocerin, one of the more recent “alternative” photoinitiators, has been developed specifically for use with bulk-fill composites [6]. The trend towards shorter clinical times for composite restorations—allowing the use of 4 mm layers as opposed to 2 mm layers of conventional composites—has given rise to bulk-fill composites.

Bulk-fill composites are the result of the trend to shorten the clinical time for composite placement by allowing placement in 4 mm layers, as opposed to 2 mm layers for conventional composites. Bulk-fill composite resins have been developed to facilitate this procedure as they allow the dentist to use thicker layers of composite materials in increments of 4–5 mm [10]. In relation to their different clinical uses, bulk-fill composite materials can be categorized as low viscosity and high viscosity [11]. The difference is visible in the method of application and their mechanical properties. Low-viscosity materials have a lower filler content, which results in lower wear resistance, i.e., they show small surface defects. They are used as a replacement for dentine tissue and require covering with a conventional composite with a minimum thickness of 2 mm. They also do not require compaction. High-viscosity materials can be used up to the level of the occlusal surface, without any need for covering [12]. 

In general, the composition of bulk-fill composite materials does not differ much from the composition of conventional composite materials. The size of the filler particles has increased, while at the same time their quantity has decreased. In the same total volume of filler, the diameter of the particles is increased to more than 20 μm, reducing the total surface of the filler particles as well as the surface of contact with the polymer matrix, which causes less scattering and deeper light penetration. 

Bulk-fill composite materials are mainly light-curing, although there are some that possess a double polymerization method [13]. Sufficient light polymerization has been accomplished by a number of compositional changes, one of which is the use of larger filler particles with correspondingly smaller specific surface areas and a lower filler volume load, which together ensure improved light transmission through the restoration material and reduced light scattering. 

Choosing resin monomers and fillers with closely matched refractive indices in unpolymerized material and adding more highly reactive photoinitiators was another compositional change. Certain bulk-fill materials contain high-molecular-weight monomers like SDR^®^ Plus Bulk Fill Flowable to reduce polymerization shrinkage, resulting in a combination of sufficient marginal adaption and deep curing [14]. But it also appears that some manufacturers have just employed bigger filler particles and less pigment to increase a material’s translucency, rather than drastically changing the material’s chemical makeup [15]. Bulk-fill composite materials possess lower shrinkage after the polymerization gelation phase and higher reactivity to light polymerization than most conventional composites as a result of their increased translucency, improving light penetration and depth of cure but they can also show variability in their colour stability when exposed to coloured drinks or food [16].

A special class of bulk-fill materials is marketed for rapid light-curing to further reduce clinical time. This group is represented by the composite Tetric PowerFill and Tetric powerFlow from Ivoclar (Schaan, Liechtenstein), which are specifically designed for high-intensity light-curing. Their composition contains chemical modifications that enable stepwise polymerization and the formation of short chains to ensure homogeneous polymerization through the 4 mm layer. Responsible for this particular behaviour is β-allyl sulphone, an addition fragmentation chain transfer (AFCT) agent, which is involved in the polymerization reaction by stopping the reaction at one polymer chain and simultaneously generating another sulfone radical that allows the polymerization reaction to continue [9,17]. An AFCT agent with a different chemistry but similar function of reducing polymerization shrinkage stress is used in Filtek™ One Bulk Fill Restorative, reported to have a higher opacity for improved aesthetics and stress relief, enabling up to 5 mm depth of cure [15].

Tetric PowerFlow (Ivoclar) is marketed as a flowable bulk-fill material for rapid light-curing. Its high translucency in the unpolymerized state and low filler content enable rapid curing though it does not contain an AFCT agent [18]. It uses Ivocerin and camphorquinone as the primary photoinitiator. For the photopolymerization of these materials, rapid curing is defined as reducing the curing time to only 3 s and increasing the irradiance of the light curing device to 3000 mW/cm^2^ using a violet–blue light source [7]. The rapid curing of Tetric PowerFill produces a degree of conversion (DC) which, according to Ilie and Watts, is comparable to that of a 10 s curing at 1500 mW/cm^2^ [19]. However, the initial polymerization rate of Tetric PowerFill is faster than that of its predecessor Tetric EvoCeram Bulk Fill (Ivoclar), which does not contain an AFCT agent. On the other hand, in vitro studies have shown that the initially faster polymerization kinetics of Tetric PowerFill and the faster development of shrinkage forces [16] neither increased polymerization stress [20] nor compromised their marginal integrity on class V cavities after thermomechanical loading [21]. The macro- and micromechanical characteristics of Tetric PowerFill showed good results throughout the 4 mm layer, but Tetric PowerFlow exhibited a higher flexural modulus in the upper 2 mm [22] than in the 2–4 mm depths [23].

These changes required modifications to the curing units used for the photoactivation of rapid-curing composites. Today, many manufacturers offer LED curing lights with a violet–blue bandwidth and an irradiance of over 3000 mW/cm^2^. LEDs are known for their long service life and are generally considered durable [24]. However, their irradiance decreases over time, albeit at a much slower rate than the previous quartz–tungsten–halogen devices. LED chips become hotter due to their increased optical power. It is well known that the optical power of LEDs decreases due to temperature stress, which ultimately shortens their lifespan [25]. 

Short time light-curing with a high-power LED curing light source can also influence temperature rise, resulting in possible pulp damage. It seems that the thin layer of adhesive could not compensate for the temperature rise, which is probably influenced by the collagen fibre structure and hybrid layer formation. It is known that heating of collagen fibres could lead to uncoupling of the triple helix structures. This occurs at a denaturation temperature that differs between the different organization levels of collagen tissue. The denaturation temperature of fully hydrated soluble collagen is near body temperature; it is considerably higher in disordered precipitated collagen (50 °C), and even higher in assembled fibrous collagen tissue (~60 °C) [26,27].

The purpose of this laboratory study was to investigate the long-term effects of rapid 3 s curing with a high-power LED curing unit on the shear bond strength of bulk-fill composite bonded to dentin using a universal adhesive and to determine the type of fracture between dentin and the composite material (adhesive, cohesive or mixed). The following null hypotheses were tested: (1) shear bond strength to dentin is not affected by the use of different polymerization protocols and (2) shear bond strength is not affected by the time periods.

## 2. Materials and Methods

This research protocol was approved by the Ethics Committee of the School of Dental Medicine University of Zagreb (No. 05-PA-30-X-9/2022), Croatia.

### 2.1. Collection and Preparation of Samples 

Human third molars (180) with fully formed roots were collected and preserved in 1% chloramine solution after extraction. The teeth were only used within three months after extraction. Only vital teeth with an intact crown and root, not damaged during extraction, without signs of periodontitis, pulp, or periapical inflammation or caries were included in this study. 

Dentin substrates were created by using a low-speed saw (IsoMet, Buehler; Lake Bluff, IL, USA) at 300 rpm with continuous water cooling. To obtain two dentin slabs, sections were taken in the mid-coronal region. An Ultradent mould (Ultradent Products, South Jordan, UT, USA) was used to mount the dentin samples using a cold-curing methacrylate resin (Technovit 4004, Kulzer, Germany). To create a flat bonding area, the dentin surface was polished with 600-grit silicon carbide (SiC) paper (PRESI, Eybenes, France), rinsed thoroughly with distilled water and instantly used for the bonding procedure.

Measurements were performed at three times (1, 6, and 12 months). For each time, a total of 120 specimens were prepared and assigned to 8 experimental groups (4 bulk-fill composite materials, 2 curing modes). The number of specimens per experimental group was *n* = 15, taking care that only one section from each tooth was in the same experimental group. One adhesive and four bulk-fill composites were used in this study (Table 1). After the slabs were split into test groups, the dentin surface was carefully air-dried until no more wetness was visible. The bonding area was demarcated with a polymer adhesive strip, which had a hole with a diameter of 2.4 mm and a thickness of 0.2 mm. The universal adhesive Scotchbond Universal Plus (3M ESPE, St. Paul, MN, USA) was used in self-etch mode with constant agitation for 20 s. A direct, gentle stream of air was then passed over the liquid for 5 s until a shiny film was formed that no longer moved in the air stream. The adhesive was light-cured for 10 s at 1000 mW/cm^2^ using Bluephase PowerCure (Ivoclar, Schaan, Liechtenstein). 

Utilizing a bonding clamp and plastic mould inserts (Ultradent Products, South Jordan, UT, USA), composite cylinders (2.38 mm internal diameter and 4.0 mm height) were created on the adhering surface in a single layer. The composite materials used in this study are described in Table 1: two materials designed for rapid curing (Tetric PowerFill and Tetric PowerFlow, both Ivoclar) and two control bulk-fill materials (Filtek One Bulk Fill Restorative, 3M ESPE, St. Paul, MN, USA, and SDR Plus Bulk Fill Flowable, Dentsply Caulk, Milford, CT, USA). The composite was light cured for 3 s (3000 mW/cm^2^) or 20 s (1000 mW/cm^2^). The LED intensity was measured using a radiometer Bluephase Meter II (Ivoclar). 

The samples were dark stored in distilled water at 37 °C for 1, 6 and 12 months before being fractured in shear mode (UltraTester, Ultradent Products) with a crosshead speed of 1 mm/min. The shear bond strength values of the adhesives to dentin were regulated in accordance with ISO 29022 [28].

The fractured composite fragments were examined with optical magnifying glasses with 3.6× magnification (Carl Zeiss Meditec AG, Oberkochen, Germany) to determine the type of fracture, i.e., the reason for the failure and the representative images of specimens with different types of fractures were photographed under a Dino-Lite digital microscope (Dunwell Tech, Inc., Torrance, CA, USA). If the fracture line was between the tooth and the composite cylinder, the fracture mode was labelled as adhesive. The fracture mode was categorised as mixed if the fracture line was partially along the adhesive interface and penetrated one of the substrates. If more than 75% of the bonding surface involved either the dentin or the composite, the fracture mode was categorised as cohesive.

### 2.2. Statistical Analysis

After verifying the normality of data distribution using the Shapiro–Wilk’s test, a three-way ANOVA was used to compare bond strength values among the materials, curing protocols, and time points. Due to statistically significant interactions among these factors, three-way ANOVA was followed by separate one-way ANOVAs performed to test for the influence of each individual factor at fixed levels of the remaining two factors. Post hoc comparisons were performed using Tukey’s adjustment, with an overall level of significance of 0.05. The statistical analysis was performed using SPSS 25.0 (IBM, Armonk, NY, USA).

## 3. Results

**Figure 1:**
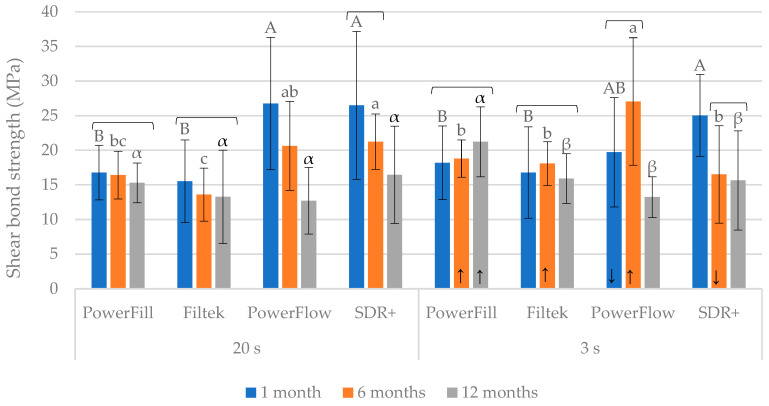
Bond strength (mean values ± 1 standard deviation) measured after 1, 6, and 12 months. Statistically homogeneous groups within each curing protocol (20 s or 3 s) are denoted by the same uppercase, lowercase, and Greek letters for 1, 6, and 12 months, respectively. Square brackets indicate statistically similar values among the time points. The upward/downward arrows within the right panel (“3 s”) indicate which values were significantly higher/lower compared to the corresponding values measured using the “20 s” curing protocol.

The bond strength values are summarized in Figure 1. Within the “20 s” curing protocol, the flowable composites, PowerFlow and SDR+, initially showed significantly higher values than the sculptable composites, PowerFill and Filtek. At the subsequent two time points, the differences among the materials within the “20 s” curing protocol were less pronounced, and finally became statistically similar for all materials after 12 months.

Within the “3 s” curing protocol, SDR+ showed significantly higher initial (1 month) values than PowerFill and Filtek. The inter-material comparisons for the “3 s” curing protocol after 6 months and 12 months mostly showed statistically similar values, except for PowerFlow (significantly higher than the other materials after 6 months) and PowerFill (significantly higher than other materials after 12 months).

The bond strength degradation was most pronounced for PowerFlow cured for 20 s, as a statistically significant decrease was identified among all three time points. Additionally, a statistically significant decrease in bond strength was observed after 6 months (SDR+ cured for 3 s) and after 12 months (SDR+ cured for 20 s and PowerFlow cured for 3 s).

The comparisons between the “20 s” and “3 s” curing protocols gave mixed results, with the “3 s” protocol showing significantly higher values in some cases (PowerFill after 6 and 12 months, Filtek after 6 months, and PowerFlow after 6 months) but also significantly lower values in other cases (PowerFlow after 1 month and SDR+ after 6 months).

**Figure 2:**
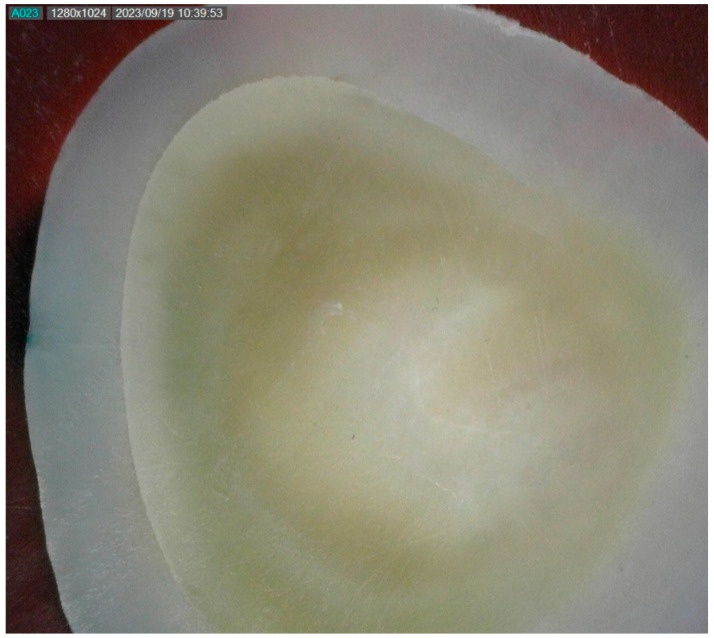
Image of a specimen with adhesion fracture under a Dino-Lite digital microscope.

**Figure 3:**
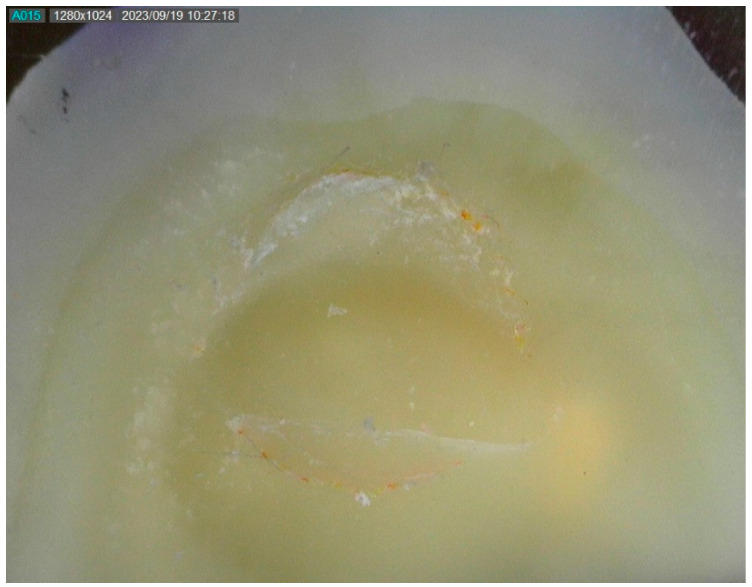
Image of a specimen with mixed fracture under a Dino-Lite digital microscope.

**Figure 4:**
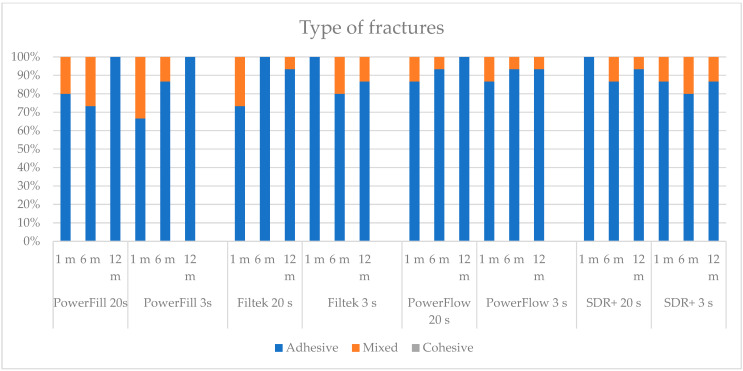
The type of fractures at the dentine–adhesive–composite interface. No cohesive fractures were detected in this study.

Two types of fractures were identified in this study, adhesive and mixed (Figure 2 and Figure 3). There were no cohesive fractures, either in the dentine or in the composite. Adhesive fractures were dominant in most experimental groups, as depicted in Figure 4. Some groups showed exclusively adhesive fractures: Tetric PowerFill at 12 months (both “3 s” and “20 s”), Filtek at 6 months cured by the “20 s” protocol and at 1 month with the “3 s” protocol, Tetric PowerFlow at 12 months cured with the “20 s” and SDR+ at 1 month cured with the “20 s” curing protocol.

## 4. Discussion

This is the first study to investigate the long-term bond strength of bulk-fill composites cured with the rapid 3 s curing protocol over a 12-month period. Two of the four materials tested were specially developed for rapid curing: the sculptable Tetric PowerFill and the flowable Tetric PowerFlow. The results showed a material-dependent response to “3 s” curing, with most materials showing the same or higher bond strength with “3 s” curing than with “standard” curing of 20 s. Therefore, the first null hypothesis is partially rejected. The initial bond strengths of the flowable composites PowerFlow and SDR+ were significantly higher than those of the sculptable composites PowerFill and Filtek during both rapid and standard curing but decreased after 12 months. In contrast, the sculptable materials showed the highest stability, as indicated by the consistent bond strength over a period of 1, 6 and 12 months for both curing protocols. After 12 months, there was no difference between the bond strengths of the two materials tested for the “20 s” group and the “3 s” group, with the exception of Tetric PowerFill, which for the “3 s” protocol showed the best long-term performance after 12 months.

The main objective of the present study was to compare the “3 s” rapid curing with “standard” 20 s curing at 1000 mW/cm^2^. Our experimental setup resulted in a radiation exposure of 9 J/cm^2^ for the 3 s curing and 20 J/cm^2^ for the standard curing. However, more than doubling the number of photons per sample surface did not result in poorer bond strength for most of the materials tested, except for PowerFlow after 1 month and SDR+ after 6 months. Amaral et al. also found that the light source and mode did not affect the microtensile bond strength, suggesting that material composition could have more influence [29]. 

The transmission of photons from the light source through a 4 mm composite layer is inevitably reduced, either by reflection at the surface, scattering by filler particles or absorption by the photoinitiators [30]. Therefore, only a limited number of photons reach the interface between the adhesive and the composite [31], where they initiate polymerisation with the superficial oxygen-inhibited layer of the adhesive and form covalent bonds between the adhesive and composite. However, it should be noted that polymerisation can be initiated in the regions closer to the light source and can continue in the deeper regions, albeit to a lesser extent. This leads to a gradual decrease in the DC and cross-linking density of the polymer network with increasing depth [32]. 

The poorer initial adhesion of PowerFlow could be related to the lower DC when this material is used with “3 s” curing compared to “20 s” curing [20], especially at 4 mm. Previous results show that bulk-fill materials with a lower DC immediately after light curing tend to show a greater increase in the DC after curing [30]. Combined with the storage temperature of 37 °C, which mobilises residual unreacted methacrylate groups [33], a post-cure increase in DC could be a possible explanation for the higher bond strength values of PowerFlow after 6 months when cured with the “3 s” curing protocol compared to “20 s” protocol. However, due to the high variability of the data, there was no statistical difference between the bond strength of PowerFlow after 1 and 6 months in the “3 s” curing regime. Nevertheless, this is only a speculation and the true causes of these differences lie beyond the current investigation. 

On the other hand, stronger bond strength with the “3 s” curing compared to the “20 s” curing of PowerFill after 6 and 12 months could be well explained. PowerFill was specially developed for polymerisation with a high-energy light in just 3 s. This development is based on two components: the AFCT agent β-allyl sulfone and the germanium-based photoinitiator Ivocerin [8,17,18]. While Ivocerin splits into two free radicals when exposed to violet light, β-allyl sulfone stops the growth of a polymer chain and simultaneously generates another free radical that can form a new initiation nucleus for the formation of another polymer chain. Thus, theoretically, three times more free radicals are available to trigger polymerization in a short 3 s, in contrast to the camphorquinone-based composites where only one radical is formed per photoinitiator molecule. It is reported that such formation of short polymer chains leads to a more homogeneous network without the gradient normally occurring in the DC [11,34]. 

Dentin bond strength decreases significantly over time in an aqueous environment [35]. The adhesive used in this study, Scotchbond Universal Adhesive Plus, has a mildly acidic pH of 2.7 and contains 10-methacryloyloxydecyl dihydrogen phosphate (10-MDP) and polyalkenoic acid copolymer to provide a chemical bond to dentin through the formation of 10-MDP calcium salts with calcium ions from dentin [36]. In contrast to one-bottle self-etching adhesives (without the additional hydrophobic layer), adhesives with water-insoluble 10-MDP–Ca salts show good long-term durability [36,37]. The differences in bond strength between the different types of adhesives are generally not apparent in the first 3 months, but in periods longer than 6 months, 10-MDP-based adhesives have demonstrated their resistance to hydrolytic degradation [38]. In the present study, the durability of bond strength with a 10-MDP-based adhesive was preserved. However, the differences among test groups were not determined by the adhesive, but by the physical properties of the composites. 

It is worth noting that the deterioration in bond strength was most pronounced in the flowable composites after 12 months, but not in the sculptable composites. Therefore, the second null hypothesis that the bond strength does not change over time is partially rejected. Like most flowable composites, both SDR+ and PowerFlow have a similarly low filler content of 47.4% and 46.4% by volume, respectively. A higher filler content is usually associated with better physical properties [39] and also with a higher bond strength [40]. A higher proportion of resin matrix than in sculptable materials also implies that these materials may be more susceptible to hydrolytic degradation, depending on the DC and the degree of crosslinking of the polymer network [41]. 

Klaric et al. investigated the water sorption and solubility for “3 s” rapid curing, using the same materials as in the present study [42]. Their results showed higher solubility for all materials in the 3 s curing protocol compared to the 20 s curing protocol. Both SDR+ and PowerFlow reached complete water saturation after 7–14 days, but also had high solubility [43]. When constantly exposed to the water environment for 12 months, there is sufficient time for the permeation of the material with water and the subsequent hydrolytic degradation of the ester groups in the resin matrix. This effect is most pronounced at the interface between the adhesive and the composite, where the composite likely has the lowest DC [32]. Therefore, the bond strength in flowable composites used in this study deteriorated considerably.

On the other hand, Filtek as the sculptable material with the highest filler volume in this study, has previously also shown the highest flexural strength, followed by PowerFill and SDR+ (which were statistically similar), and PowerFlow [43]. However, in the present study, Filtek did not exhibit the highest bond strength in any of the curing protocols. This is probably due to its low DC value, as previously shown [42,43]. The distinctive feature of this material is a long, high-molecular-weight AUDMA molecule with only two methacrylate groups [44]. Previous investigations have shown that the DC of this material significantly drops in 4 mm depth, especially if “3 s” curing is employed [43,44]. It appears that the AUDMA and high viscosity of this material slow down the polymerization kinetics, so that “3 s” curing is not efficient for Filtek [45], leaving unreacted methacrylate groups. The experimental conditions in the present study may have allowed for some post-cure DC increase in “3 s” curing of Filtek, which would have resulted in a higher bond strength than the standard “20 s” curing at the 6-month time point. 

When a composite material bonded to a flat dentin surface is sheared or tensile loaded, the stress distribution along the joint is highly irregular. The shear bond strength can be related to the modulus of elasticity of the enamel–dentin adhesion system. Increasing the modulus of elasticity will result in a more even distribution of stress over the bonded area and avoid stress concentration at the point of application of the load. In addition, studies have found a positive correlation between the shear bond strength to dentin and the flexural modulus of the composite used [46]. It has been noted that the modulus of elasticity of restorative materials increases roughly in line with the increase in shear bond strength. A stiffer composite material will result in different interfacial stress distributions and lead to an apparently higher bond strength value [47].

SDR Plus Bulk Fill Flowable and Tetric PowerFlow, the materials used in this research, are low-viscosity thick-layer composite materials that have a lower modulus of elasticity. In this study, bulk-fill materials were used primarily due to their increased translucency, i.e., facilitated penetration of light into deeper layers, which potentially leads to faster polymerization, and its smaller polymerization contraction means lower polymerization stress. According to data from the literature, compared to conventional composites, due to its lower modulus of elasticity, bulk materials can show lower bond strength values.

As concluded in the review work by Scherrer et al., a large scatter of data on the bond strength of dentin was found in the literature [48], and the overall stress distribution at the dentin–composite interface was determined not only by the load distribution, but also by sample preparation, material properties, fixation and load conditions [49]. It is recommended that comparative tests of bond strength are possible only at the level of identical combinations of enamel–dentin adhesion systems and composites, and certainly not at the level of only enamel–dentin systems [50].

Because studies of bond strength to dentin found in the literature for the materials used in this study could not be correlated due to variations in sample preparation, testing methods, and storage conditions, the data could not be adequately compared. Despite this, this study followed the recommendation to normalize the shear bond strength so that future studies conducted in the same way could be compared [51].

Care should be taken when interpreting the results of this in vitro study. One of the drawbacks of the study is that bulk-fill composites were tested after aging in distilled water. Low viscosity bulk-fill (Tetric PowerFlow and SDR) are meant to be capped clinically with a high viscosity material. The efficient time saving offered by non-selective 3 s curing being used for all bulk-fill composites is attractive for clinical practice. It should be noted that even if some of the tested properties are not affected by rapid curing, there are a number of others, such as DC or water solubility. Despite relatively positive results for long-term adhesion with rapid 3 s curing of materials not intended for such use, dentists should be cautious with such practice.

## 5. Conclusions

Under the limitations of this in vitro study, it is possible to conclude that rapid 3 s curing with a high-power LED curing unit has an effect on the shear bond strength of bulk-fill composite bonded to dentin using a universal adhesive. These results have important implications for dentists when selecting composite materials and curing protocols. Although “3 s” curing resulted in equal or better shear bond strength in the majority of conditions tested, based on the results of the present in vitro study, we can recommend its use only for material with an AFCT agent, such as Tetric PowerFill. 

## Figures and Tables

**Table 1:** The type and composition of the investigated materials according to the manufacturer’s instructions.

Material (Abbreviation)	Type	Manufacturer	Organic Matrix	Fillers (wt%/vol%)	Lot
**Scotchbond Universal Plus**	Adhesive	3M ESPE Dental Products; St. Paul, MN, USA	MDP monomer, dimethacrylate resins		8165721
**3M™ Filtek™ One Bulk Fill Restorative**	Sculptable composite	3M ESPE Dental Products; St. Paul, MN, USA	AUDMA, diurethane-DMA, 1,12-dodecan-DMA	~76.5/ ~58.5	NC09993
**Tetric^®^ PowerFill**	Sculptable composite	Ivoclar AG; Schaan, Liechtenstein	monomer matrix—dimethacrylate (wt = 20–21%)	76–77/53–54	Z009GW
**SDR^®^ Plus Bulk Fill Flowable**	Flowable composite	Dentsply Caulk; Milford, DE, USA Dentsply DeTrey GmbH; Konstanz, Germany	resin matrix—modified UDMA, TEGDMA, dimethacrylate and three methacrylate resins	70.5/47.4	2101000559
**Tetric^®^ PowerFlow**	Flowable composite	Ivoclar AG; Schaan, Liechtenstein	monomer matrix—dimethacrylate (wt = 28%)	68.2/46.4	Z00V4H

MDP: methacryloyloxydecyl dihydrogen phosphate; UDMA: urethane dimethacrylate; TEGDMA: triethylene glycol dimethacrylate; AUDMA: aromatic urethane dimethacrylate; DMA: dimethacrylate.

## Data Availability

The data that support the findings of this study are available from the corresponding author, D.M., upon reasonable request.
